# Exposure to Fumonisins and the Occurrence of Neural Tube Defects along the Texas–Mexico Border

**DOI:** 10.1289/ehp.8221

**Published:** 2005-09-29

**Authors:** Stacey A. Missmer, Lucina Suarez, Marilyn Felkner, Elaine Wang, Alfred H. Merrill, Kenneth J. Rothman, Katherine A. Hendricks

**Affiliations:** 1 Department of Epidemiology, Harvard School of Public Health, Boston, Massachusetts, USA; 2 Department of Obstetrics, Gynecology, and Reproductive Biology, Brigham and Women’s Hospital and Harvard Medical School, Boston, Massachusetts, USA; 3 Texas Department of State Health Services, Austin, Texas, USA; 4 School of Biology, Georgia Institute of Technology, Atlanta, Georgia, USA; 5 Department of Epidemiology, Boston University School of Public Health, Boston, Massachusetts, USA; 6 Medical Institute, Austin, Texas, USA

**Keywords:** case–control study, corn, fumonisins, Mexican Americans, mycotoxins, neural tube defects

## Abstract

Along the Texas–Mexico border, the prevalence of neural tube defects (NTDs) among Mexican-American women doubled during 1990–1991. The human outbreak began during the same crop year as epizootics attributed to exposure to fumonisin, a mycotoxin that often contaminates corn. Because Mexican Americans in Texas consume large quantities of corn, primarily in the form of tortillas, they may be exposed to high levels of fumonisins. We examined whether or not maternal exposure to fumonisins increases the risk of NTDs in offspring using a population-based case–control study. We estimated fumonisin exposure from a postpartum sphinganine:sphingosine (sa:so) ratio, a biomarker for fumonisin exposure measured in maternal serum, and from maternal recall of periconceptional corn tortilla intake. After adjusting for confounders, moderate (301–400) compared with low (≤ 100) consumption of tortillas during the first trimester was associated with increased odds ratios (ORs) of having an NTD-affected pregnancy (OR = 2.4; 95% confidence interval, 1.1–5.3). No increased risks were observed at intakes higher than 400 tortillas (OR = 0.8 for 401–800, OR = 1.0 for > 800). Based on the postpartum sa:so ratio, increasing levels of fumonisin exposure were associated with increasing ORs for NTD occurrences, except for the highest exposure category (sa:so > 0.35). Our findings suggest that fumonisin exposure increases the risk of NTD, proportionate to dose, up to a threshold level, at which point fetal death may be more likely to occur. These results also call for population studies that can more directly measure individual fumonisin intakes and assess effects on the developing embryo.

In 1990–1991, an outbreak of neural tube defects (NTDs) occurred in Cameron County, Texas (USA), a south Texas county bordering Mexico wherein six anencephalic births occurred in 6 weeks at one hospital. NTDs are embryonic defects of the brain and spinal cord resulting from failure of the neural tube to close. Spina bifida and anencephaly (failure of anterior tube closure) are the most common forms of NTD. Investigation of the cluster revealed a high prevalence of NTDs in this region (27 per 10,000 live births) (Texas Department of Health, unpublished report) that proved endemic to the entire Texas–Mexico border region. Just before the NTD outbreak, in the fall of 1989, an outbreak of equine leukoencephalomalacia (ELEM)—liquefaction of the white matter of the brain in horses—occurred nationwide. It was particularly severe in Texas, where, in contrast to the usual one to five ELEM clusters reported, > 40 clusters were reported in < 2 months (Reagor J, personal communication). As an empiric preventive measure, livestock were taken off feed containing corn, because the corn harvest that year was thought to be contaminated with mold (Ross et al. 1992). Humans continued to eat corn from this harvest. The corn contaminant that led to the ELEM epizootic was a class of mycotoxins called fumonisins, produced by the molds *Fusarium verticillioides* (sometimes and formerly referred to as *Fusarium moniliforme*) (Seifert et al. 2003).

Multiple observations suggested that the NTD outbreak and the epizootics shared a common etiology. Cornmeal samples collected in the United States during the NTD outbreak had relatively high average fumonisin levels (Hendricks 1999). Other regions with high corn-based food consumption and documented fumonisin contamination (Dombrink-Kurtzman and Dvorak 1999; Yoshizawa et al. 1994) also have high prevalences of NTDs (Moore et al. 1997; Mutchinick et al. 1999). Recent *in vitro* and animal studies provide further support for the hypothesis that NTDs occur with exposure to fumonisins (Flynn et al. 1997; Gelineau-van Waes et al. 2005 ;Sadler et al. 2002; Stevens and Tang 1997; Wang et al. 1991).

In this study, we examined whether or not maternal exposure to fumonisins increases the risk of NTDs in offspring. This examination, using a population-based case–control study, represents the first epidemiologic assessment of the association of NTD occurrence and fumonisin exposure. We estimated fumonisin exposure from postpartum sphinganine:sphingosine (sa:so) ratio, a biomarker for fumonisin exposure measured in maternal serum, and from maternal recall of periconceptional corn tortilla intake.

## Materials and Methods

### Study population.

We identified study participants through the Texas Department of Health’s Neural Tube Defect Project. Participant identification and data collection methods have been described previously in detail (Hendricks et al. 1999; Suarez et al 2000). In brief, the project included multi-source active surveillance, a case–control study to identify risk factors for NTD occurrence, and a follow-up folic acid intervention program to reduce NTD recurrence. Cases (infants or fetuses) had a diagnosis of anencephalus [*International Classification of Diseases, 9th Revision Clinical Modification* (ICD-9-CM) (Medicode, Inc. 1993)] code 740, spina bifida (741), or encephalocele (742.0) and included live births, stillborns, and prenatally diagnosed fetuses at all gestational ages. For this study, we defined case women as Mexican-American women with NTD-affected pregnancies who resided and delivered in one of the 14 Texas–Mexico border counties from March 1995 through May 2000. We identified control women from Mexican-American residents of the same study area who delivered normal live births during the same period. Control women were randomly selected annually, frequency matched to case women by facility and year. Facilities included hospitals and midwife-attended birthing centers.

### Data collection.

The Texas Department of Health Institutional Review Board approved the study design, English–Spanish consent forms, English–Spanish interview instruments, specimen collection, and study procedures. In cooperation with hospital staff, field teams contacted women at the time of delivery or termination of pregnancy to inform them of the study and obtain consent. All women in the study gave written informed consent, in their preferred language (English or Spanish), before participation in the study.

Women were interviewed in person about 5–6 weeks postpartum with an interview instrument modeled after the 1993 Centers for Disease Control and Prevention (CDC) mother questionnaire for birth defects risk factor surveillance. The questionnaire assessed maternal health history, demographics, use of medications and nutritional supplements, and environmental and occupational exposures during the periconceptional period—the 3 months before conception and the 3 months after conception (first trimester of pregnancy). Before each interview, the staff obstetrician/gynecologist and interviewer estimated the date of conception for the index pregnancy using all gestational age estimates from the medical records. Women were specifically asked about corn tortilla consumption during periconception, including the type (brand name, homemade), the month consumed, the frequency (number of days per month), and quantity (number of tortillas per day). We calculated body mass index (BMI; kilograms ÷ meter^2^) from self-reported prepregnancy height and weight. Women were paid $20 for the 2-hr interview. Blood and urine samples were also collected, for which women were paid an additional $20.

### Laboratory procedures.

Maternal blood specimens were collected in 13 mL tubes without anticoagulant. After coagulation and centrifugation, 3 mL aliquots of serum were apportioned into cryovials. These aliquots were frozen at 20°C and shipped overnight on dry ice to Emory University Laboratory (Atlanta, GA).

Analysis of the sa:so ratio in serum was conducted by high-performance liquid chromatography with fluorescent detection of these compounds as the *ortho*-phthalaldehyde derivatives (Riley et al. 1994). Fumonisins inhibit ceramide synthase, which results in an elevation of sphinganine, a potentially toxic intermediate of *de novo* sphingolipid biosynthesis (Merrill et al. 2001; Riley et al. 2001; Wang et al. 1991). Because sphingosine is formed during turnover of complex sphingolipids, its amount is affected less by fumonisins; hence, the sa:so ratio is a surrogate measure of fumonisin exposure. Owing to funding restrictions, the laboratory conducted the sphinganine and sphingosine analyses in two separate batches, each containing case and control samples. The Division of Laboratory Sciences of the CDC performed serum folate and B_12_ analyses using the same procedure as that used for the Third National Health and Nutrition Examination Survey (Gunter et al. 1996). The long-term total analytical coefficient of variation for these assays over 6 years was approximately 5%.

To estimate fumonisin levels in tortillas, we collected samples of tortillas from participant homes and local grocery stores throughout the study period. A total of 146 households contributed tortilla samples, and another 114 samples were obtained from grocery stores. Tortillas were placed in plastic reclosable sandwich bags and labeled with the date of collection, brand of tortilla, and place of tortilla purchase. The samples were frozen at 0°C until shipped, and then shipped in cold packs overnight to the Division of Natural Products laboratory at the U.S. Food and Drug Administration (FDA). Of the multiple structural isoforms of fumonisin B_1_ (FB_1_), fumonisin B_2_ (FB_2_), fumonisin B_3_ (FB_3_), we report only FB_1_ levels; FB_2_ and FB_3_ levels were essentially nondetectable. FB_1_ levels were determined using high-pressure liquid chromatography (Stack 1998).

### Statistical analysis.

Of the 225 Mexican-American women with NTD-affected pregnancies and 378 Mexican-American women with healthy live births identified for study, 184 case women and 225 control women participated in the interview. Twenty-six case women (12%) and 101 control women (27%) refused to be interviewed, and 15 case women (7%) and 52 control women (14%) had moved out of the study area without being interviewed. Of those interviewed, 163 case women (89%) and 189 control women (84%) provided blood specimens for the sa:so assay.

We evaluated two direct exposure metrics: fumonisin exposure as measured by the sa:so ratio assayed from the maternal blood sample, and total number of corn tortillas eaten during the first trimester of pregnancy as reported on the mothers’ questionnaires. We chose categorical cut-points for presentation of the fumonisins and continuous corn exposure variables by calculating the effect of finely categorized variables and then coalescing adjacent categories based on the observed effect estimates (Greenland and Rothman 1998).

In addition, we calculated a third metric based partially on ecologic data: nanograms of fumonisins ingested per day during the periconceptional period as estimated from grouped 6-month averages for the tortilla samples. To control for possible seasonal variability, we categorized dates of conception into 6-month blocks, beginning with February–July 1994 and ending with August 1999–January 2000. We chose these categories based upon the timing of the entry of new corn crops into the U.S. market for human purchase and consumption (Miller D, personal communication). We linked each woman’s date of conception to the average fumonisin levels found in tortillas collected during that 6-month block. Daily dose of fumonisin exposure was calculated by multiplying the average fumonisin level (nanograms per gram) by 24 g per tortilla (the average weight of collected samples) and then by the number of tortillas the woman reported eating per day during the first trimester of pregnancy. That number was then divided by the woman’s weight in kilograms.

Using the SAS statistical software package (Version 8, SAS Institute 1991), we fit a logistic regression to calculate deconfounded odds ratios (ORs) and 95% confidence intervals (CIs) to estimate prevalence ratios of NTDs. We considered other possible risk factors for NTDs as potential confounders if addition of that variable to the model changed the OR by 10% or greater. Confounding checks were performed in both univariate and final multivariate models. If a factor was identified as a confounder of any estimated main effect, it was kept in all models. Based on these criteria, we controlled for BMI (kilograms per square meter, continuous), serum B_12_ (picograms per milliliter, continuous), and dates of conception (6-month blocks). In addition, the sa:so values were found to differ systematically by batch (batch 1: *n* = 172, ratio median = 0.19, 5th percentile = 0.08, 95th percentile = 0.36; batch 2: *n* = 180, ratio median = 0.13, 5th percentile = 0.08, 95th percentile = 0.24), and therefore we adjusted for batch in all sa:so analyses. Known NTD risk factors that were not confounders within this population included maternal age, maternal birthplace, annual household income, folate intake (dietary plus multivitamin), and prior pregnancy loss. Finally, because it is unclear whether serum folate levels are a measure of an intermediate variable between the main corn/fumonisin effects and NTDs or whether serum folate is a potential confounder, we fit models with and without adjustment for serum folate.

## Results

The demographic profile of control women illustrates the low socioeconomic conditions prevalent on the border. More than a third reported a household income of < $10,000 per year, and only about half had completed high school (Table 1). Control women were leaner and more educated than were case women but comparable in age. Few women reported ever taking preconceptional folic acid supplements.

Nearly all women consumed corn tortillas (96% of case women and 93% of controls). As seen in Table 2, control women reported an average consumption of two tortillas per day during their first trimester of pregnancy (180 total during this 3-month period). That number was nearly identical to the intake reported during the 3 months before conception. Most women purchased rather than made corn tortillas.

After adjustment for BMI, serum B_12_, and date of conception, moderate (301–400) compared with low (≤ 100) consumption of tortillas during the first trimester was associated with an increased OR of having an NTD-affected pregnancy (OR = 2.4, 95% CI, 1.1–5.3) (Table 3). Higher intakes (401–800 and > 800) were associated with either a slight decrease in occurrence (OR = 0.8 for 401–800) or no effect (OR = 1.0 for > 800). The type of tortilla usually consumed appeared to affect risk. NTD risk increased with exposure to homemade tortillas (OR = 2.9; 95% CI, 1.4–5.9) (Table 3). Among those who purchased tortillas, the effect estimates differed slightly by brand, but the CIs for individual brands were wide (data not shown). As shown in Table 3, increased exposure to fumonisins, based on postpartum sa:so ratio, was associated with an increased NTD occurrence except for the highest exposure category. The highest exposure category (sa:so > 0.35) was related to less frequent occurrence (OR = 0.7, 95% CI, 0.2–2.9) but was based on the fewest number of subjects and therefore had a wide CI. Results differed negligibly when serum folate was added to the model.

The mean fumonisin level measured in the 240 tortilla samples was 234 ng/g (range = 0–1,690 ng/g; SD = 256). When fumonisin levels by season were linked to dates of conception, the median daily fumonisin exposure (nanograms per day per kilogram of weight) was 172.6 for case women and 156.1 for control women. Using the imputed exposure levels for individual women, we also observed an inverted U-shape relation to risk, reflecting the tortilla consumption pattern (Table 4).

## Discussion

Our findings from the postpartum serum sa:so measure suggest that fumonisin exposure increases the risk of NTD, proportionate to dose, up to a threshold level. Data on the corn tortilla consumption and fumonisin ingestion appear to support an inverted U-shaped pattern of occurrence. This pattern may reflect the formation of NTDs up to a threshold of damage, at which point fetal death is more likely to occur. Because true biologic incidence is impossible to determine, the prevalence of birth defects is a function of embryologic incidence as well as intrauterine survival (Weinberg and Wilcox 1998). Additionally, experiments demonstrate that fetal resorption can occur in folate-deficient pregnant mice (Burgoon et al. 2002) as well as in hamsters and mice exposed to high doses of FB_1_ (Floss et al. 1994; Gelineau-van Waes et al. 2005; Reddy et al. 1996).

We also observed a difference in risk effect between manufactured tortillas and homemade tortillas. The variations in small-scale tortilla preparation, especially the corn-to-lime ratio, results in wide variations in residual fumonisins (De La Campa et al. 2004). If tortillas made at home have a consistently lower concentration of lime or poorer quality corn is used, this could potentially explain some of the increased effect seen in homemade tortillas (De La Campa et al. 2004).

Alternative explanations for the effects that we observed include concern about the potential for recall bias in the estimate of tortillas consumed and lack of a true biomarker of fumonisins at the time of neural tube closure. Case and control women were asked to recall corn consumption as much as a year earlier. However, dietary recall over a much longer period has been shown to be generally reliable (Byers et al. 1987). Although it might seem intuitive that case women were more motivated to remember some events as they sought explanations for having a child with a birth defect, this has not proved to be a consistent bias in studies comparing prospective and retrospective measurement of exposures (Khoury et al. 1994; Mackenzie and Lippman 1989). It seems unlikely that an event as routine as eating corn tortillas would have been differentially recalled. Furthermore, recall bias would not easily account for the consistent observation of an inverted U-shaped relation between questionnaire and laboratory-measured exposures.

Temporality of the blood sample collection is also an important concern. The sa:so ratio measures acute exposure to fumonisins, with levels returning to normal when the exposure is removed. In our sample, the sa:so ratio was measured 5–6 weeks postpartum and would reflect periconceptional levels at neural tube closure only if study participants were exposed to fumonisins at a constant level. We note, however, that in rats and mice, a subtoxic fumonisin dose will maintain elevated sphinganine when it follows exposure to a higher dose (Enongene et al. 2002; Wang et al. 1999). We have observed that tortilla consumption varies little with women’s pregnancy status, making it possible that this population is continuously exposed to some level of fumonisins. In addition, the biochemical mode of action gives no indication that this is a case of reverse causation, that is, the NTD (due to low folate or the shorter gestation) causing elevated sa:so ratios in the mother (Gelineau-van Waes et al. 2005).

Our imputation of daily fumonisin consumption also had several limitations. To provide an estimate of fumonisin consumption from corn tortillas, tortillas were collected from homes and local grocery stores, which may or may not indicate what was actually consumed by each woman. In addition, the collection of corn tortillas was not systematic, and the samples were not proportionately representative of region, brand, or season. Further, the daily dose of fumonisin exposure depended on the number of tortillas women recalled, which as we noted is subject to error. Despite these limitations, it is noteworthy that the estimated levels of fumonisin ingestion for some women (650–9,441 ng/kg body weight for women in the highest quartile) surpass the World Health Organization’s maximum tolerable daily intake of 2 μg/kg (2,000 ng/kg) of body weight of any combination of fumonisins (WHO 2002).

The higher proportion of refusals to participate among control women (27%) compared with case women (12%) is another potential concern. Analyses conducted by Suarez et al. (2000) validated that control women mirrored the demographic characteristics of all border Mexican-American women who gave birth during the study years, indicating the low likelihood that control women were seriously unrepresentative of the border population.

The evidence that fumonisins may play a role in the development of NTDs is slowly being assembled. Through our epidemiologic study, we have documented that a population with a high prevalence of NTDs also consumes large amounts of corn products (tortillas). In addition, we have demonstrated that the amount of fumonisins in corn used to make tortillas may be high. We have illustrated with laboratory data that, within a certain range, women with increasing sa:so ratios (a surrogate for fumonisin exposure) are increasingly likely to have had an NTD-affected pregnancy, independent of known NTD risk factors (folate, B_12_, obesity, and other covariates). Previous work shows that this population obtains folate largely through diet and that the use of folic acid–containing vitamins is rare. The negligible effect of serum folate in the model reflects this lack of supplementation (Suarez et al. 2000). In fact, B_12_ levels are a more important predictor of NTD risk than are folate levels in this population (Suarez et al. 2003), a risk factor included in the adjusted model for fumonisin exposure.

Recent laboratory experiments complement these epidemiologic findings by illuminating the biologic mechanisms through which fumonisins might increase risk for NTDs. Fumonisins have been shown to inhibit the biosynthesis of sphingolipids (Wang et al. 1991), which interferes with the uptake of 5-methyltetrahydrofolate and decreases total folate binding (Stevens and Tang 1997). Furthermore, mice embryos subjected to fumonisin exposure develop NTDs *in vitro* (Flynn et al. 1997; Sadler et al. 2002) and *in vivo* (Gelineau-van Waes et al. 2005). Administration of folate reverses this effect (Gelineau-van Waes et al. 2005; Sadler et al. 2002). Cumulatively, these biologic and epidemiologic findings support the hypothesis that fumonisin contamination of corn destined for human consumption poses a risk for NTDs through its impact on sphingolipid and, ultimately, folate metabolism.

Based on our findings, it is possible that the 1990–1991 Cameron County NTD outbreak shared an etiology with the ELEM epizootics that slightly preceded it. The discovery of an association between fumonisin exposure and NTDs may help to clarify both the etiologies of unexplained NTD outbreaks and the increased background prevalence observed in some populations (Moore et al. 1997; Mutchinick et al. 1999). In 2001, the FDA recommended that an evaluation of the dietary intake of corn products by specific population groups (e.g., Texas Hispanics) and the levels of fumonisins found in those corn products was needed to fully assess the potential health risks (U.S. FDA 2001). Future epidemiologic studies should focus on measuring individual fumonisin intake in specific high-risk populations and assessing its impact not only on the developing embryo but also on other outcomes such as impaired fecundity or pregnancy loss.

## Figures and Tables

**Table 1:** Distribution of selected maternal factors by NTD status.

Characteristic	Cases (*n* = 184)	Controls (*n* = 225)
Maternal age [years; median (5th, 95th^a^)]	23 (16, 35)	23 (15, 34)
Maternal education [years of school; median (5th, 95th^a^)]	11 (5, 15)	12 (6, 16)
Annual household income [*n* (%)]		
≤ $10,000	81 (44.0)	80 (35.6)
> $10,000 to ≤ $15,000	35 (19.0)	47 (20.9)
> $15,000 to ≤ $20,000	18 (9.8)	28 (12.4)
> $20,000 to ≤ $25,000	13 (7.1)	22 (9.8)
> $25,000	32 (17.4)	46 (20.4)
Missing	5 (2.7)	2 (0.9)
Maternal BMI [kg/m^2^; median (5th, 95th^a^)]	25.6 (18.8, 39.7)	24.6 (18.3, 34.5)
Took preconceptional folic acid supplements [*n* (%)]	11 (6.0)	10 (4.4)
Serum B_12_ (ng/mL)		
Median (5th, 95th^a^)	429 (222, 935)	497 (280, 1,038)
Missing [*n* (%)]	27 (14.7)	38 (16.9)
Serum folate (ng/mL)		
Median (5th, 95th^a^)	11.3 (2.4, 39.6)	11.4 (3.4, 32.2)
Missing [*n* (%)]	26 (14.1)	37 (16.4)

a Percentiles.

**Table 2:** Distribution of corn-related exposures in women by NTD status.

Characteristic	Cases (*n* = 184)	Controls (*n* = 225)
No. of tortillas eaten during the 3 months before conception		
Median (5th, 95th^a^)	261 (17, 900)	180 (12, 900)
Missing [*n* (%)]	24 (13.0)	21 (9.3)
No. of tortillas eaten during the first trimester		
Median (5th, 95th^a^)	252 (15, 900)	180 (12, 900)
Missing [*n* (%)]	25 (13.6)	23 (10.2)
Source of corn tortillas [*n* (%)]		
Purchased	144 (78.3)	192 (85.3)
Homemade	4 (2.2)	4 (1.8)
Both	23 (12.5)	14 (6.2)
Missing	13 (7.1)	15 (6.7)
Serum sa:so ratio		
Median (5th, 95th^a^)	0.16 (0.08, 0.33)	0.14 (0.07, 0.35)
Missing [*n* (%)]	21 (11.4)	36 (16.0)

a Percentiles.

**Table 3:** Associations between corn-related exposures and the OR of NTDs.

Characteristic	Cases	Controls	Crude OR (95% CI)	Adjusted OR^a^ (95% CI)
Ate any tortillas				
No	8	15	1.0 (referent)	1.0 (referent)
Yes	176	210	1.6 (0.7–3.8)	2.3 (0.8–6.8)
No. of tortillas eaten during the first trimester				
≤ 100	55	66	1.0 (referent)	1.0 (referent)
101–300	35	73	0.5 (0.3–0.9)	0.6 (0.3–1.0)
301–400	27	17	1.8 (0.9–3.5)	2.4 (1.1–5.3)
401–800	28	29	1.1 (0.6–2.0)	0.8 (0.4–1.6)
> 800	14	17	0.9 (0.4–2.0)	1.0 (0.5–2.3)
Source of tortillas				
Never homemade	144	192	1.0 (referent)	1.0 (referent)
Ever homemade	27	18	2.0 (1.1–3.8)	2.9 (1.4–5.9)
Serum sa:so ratio				
≤ 0.10	19	36	1.0 (referent)	1.0 (referent)
0.11–0.15	51	65	1.6 (0.8–3.1)	1.5 (0.8–3.0)
0.16–0.20	41	41	2.0 (1.0–4.2)	2.0 (1.0–4.2)
0.21–0.25	23	20	2.4 (1.0–5.8)	2.4 (1.0–5.7)
0.26–0.30	15	13	2.5 (0.9–6.7)	2.4 (0.9–6.6)
0.31–0.35	11	5	4.5 (1.3–15.8)	4.4 (1.2–15.5)
> 0.35	3	9	0.7 (0.2–2.8)	0.7 (0.2–2.9)

a Adjusted for BMI (kg/m^2^), serum B_12_, and date of conception (in 6-month blocks). sa:so ratio models also adjusted for blood assay batch (1 or 2).

**Table 4:** Maternal fumonisin exposure imputed from tortilla samples and OR of NTDs.

Fumonisins^a^ (ng/day/maternal kg weight)	Cases	Controls	Crude OR (95% CI)	Adjusted OR^b^ (95% CI)
≤ 30.0	13	28	1.0 (referent)	1.0 (referent)
30.1–150.0	47	63	1.6 (0.8, 3.4)	1.9 (0.9, 4.3)
150.1–650.0	58	66	1.9 (0.9, 4.0)	2.3 (1.1, 5.1)
> 650.0	19	28	1.5 (0.6, 3.5)	1.1 (0.4, 3.0)

a Fumonisins measured from tortilla samples that were assayed and linked to dates of conception.

b Adjusted for serum B_12_ only because measure is per weight and linked to dates of conception.
